# Effectiveness of a Personal Vaccination Recommendation Letter to Liver Transplant Patients and Family Physicians for Improving the Vaccination Status: Improving Vaccinations: Whom to Contact?

**DOI:** 10.1111/ctr.70239

**Published:** 2025-09-26

**Authors:** Dorothea Dehnen, Maia Milzkott, Benjamin Borchardt, Anette Graute, Katja Dehnen, Kerstin Herzer, Katharina Willuweit, Anna Herwig, Jassin Rashidi‐Alavijeh, Birgitta Weltermann

**Affiliations:** ^1^ Institute of General Practice Medical Faculty University of Duisburg‐Essen Essen Germany; ^2^ Knappschafts‐Klinik Bad Neuenahr Bad Neuenahr‐Ahrweiler Germany; ^3^ Department of Gastroenterology Hepatology and Transplant Medicine Medical Faculty University Hospital Essen, University of Duisburg‐Essen Essen Germany; ^4^ Institute of General Practice and Family Medicine University Hospital Bonn, University of Bonn Bonn Germany

**Keywords:** family physicians, liver transplantation, randomized intervention study, vaccination

## Abstract

**Background:**

Vaccination rates of immunosuppressed liver transplant recipients need to improve. We compared the effectiveness of a three‐arm randomized intervention on vaccination rates in liver transplant recipients.

**Methods:**

Two hundred and eighty‐nine liver transplant recipients were randomly assigned to three groups: (1) patient intervention, (2) family physician intervention, and (3) combined patient and family physician intervention. The intervention consisted of a yellow information letter, which was personalized and sent to either the patient (1), the family physician (2), or both (3) in December 2016.

**Results:**

Irrespective of the assigned intervention group, a significant increase in vaccination rates from baseline to follow‐up after 2 years was observed for tetanus (53%–56%), pertussis (52%–57%), hepatitis A (44%–49%), hepatitis B (64%–71%), pneumococci (68%–75%), and the sequential pneumococcal vaccination (12%–22%). Comparing the interventions, the vaccination rate for hepatitis A was significantly higher at follow‐up for intervention (3) (OR = 9.07, *p* = 0.043) and (2) (OR = 9.91, *p* = 0.034) than for intervention (1). Concerning the vaccination rate for hepatitis B, an odds ratio of 9.13 was observed for intervention (2) (*p* = 0.006) compared to (1). Interventions (2) and (3) were generally superior to the patient‐centered intervention alone, with the exception of influenza vaccination.

**Conclusion:**

The results show the importance of family physicians in improving vaccination rates in liver transplant recipients. Establishing low‐threshold communication channels, for example, via the electronic patient record, could improve cooperation between physicians in specialized outpatient clinics and family physicians.

**Trial Registration:**

German Clinical Trials Register (DRKS: DRKS00035422). Registration was done retrospectively due to time constraints and lack of human resources.

**Abbreviations**
LT: liver transplant
PCV 13: 13‐valent pneumococcal conjugate vaccine
PPSV 23: 23‐valent pneumococcal polysaccharide vaccine
STIKO: Standing Commission on Vaccination
TI: telematics infrastructure

## 1 Introduction

Immunocompromised patients, such as those after organ transplantation, are at increased risk of vaccine‐preventable infections, including illnesses caused by influenza and pneumococci [1, 2, 3, 4]. Nevertheless, studies have shown that the vaccination status of this particularly vulnerable group is often inadequate [1, 5, 6, 7, 8]. In a previous study, we were able to demonstrate that the vaccination status of 444 liver transplant (LT) recipient in the liver transplantation outpatient clinic of the University Hospital Essen required significant improvement, as only 0.7% of the patients had received all recommended vaccinations [9]. Felzer et al. examined the vaccination rates of organ transplant recipients in the US, considering influencing factors such as age, socioeconomic status, race, and so on. They found that vaccination rates among solid organ transplant recipients were below national targets for influenza and pneumococci, and that vaccination rates were particularly low among patients living outside urban areas [10].

There are a variety of factors contributing to low vaccination rates, such as poor organization of vaccination management, lack of trust in vaccinations, and insufficient knowledge of the family physicians about the efficacy and timing of vaccination. For example, knowledge about the lower humoral vaccination response in immunocompromised patients, such as LT recipients, is not widespread among family physicians. Low awareness among vulnerable patients about the need to be completely vaccinated and the lack of recommendations from their family physician are also known reasons for low vaccination rates. Only a small percentage of people show hesitant or negative behavior toward vaccination [1, 9, 11, 12, 13].

Different approaches focusing on patients, physicians, and the health care system have been taken to improve vaccination rates [14, 15, 16]. According to a review of 75 studies by the Cochrane Collaboration, all types of patient reminders and recalls were effective in the included studies, and increased vaccination rates by 5%–20%. For example, telephone reminders were more effective than letter reminders, which in turn were slightly more effective than text messages, postcards, and autodialers [14]. In a survey by Sanftenberg et al., 86.3% of family physicians expressed their willingness to administer vaccinations to their patients as recommended in a discharge letter. The authors concluded that a standardized inclusion of vaccination recommendations in discharge letters can promote the involvement of clinicians in the vaccination process and at the same time remind family physicians of necessary vaccinations [17].

Based on the results of our baseline study, we showed that enhanced communication with the family physicians through information letters included in the outpatient clinic discharge letter and telephone calls to patients improved the number of vaccinations. The pneumococcal vaccination rate (≥1 during lifetime), for example, increased from 62.8% to 76.3% [18]. Nevertheless, there was further potential for improvement, especially with regard to the annual influenza vaccination (mean value for 2014–2016: 25%) [18]. Evidence of a benefit from newer approaches such as the use of apps is currently pending. For example, Feldmann et al. are currently investigating the benefits of an app that reminds both parents of pediatric patients awaiting transplantation and their treating physicians of necessary vaccinations [19].

In Germany, family physicians are mainly responsible for administering vaccinations [17, 20]. Health insurance funds conclude contracts with outpatient doctors organized in the Association of Statutory Health Insurance Physicians (*Kassenärztliche Vereinigung*) for billing services. Hospital doctors must obtain authorization to provide services under statutory health insurance in order to bill health insurance funds for outpatient services. However, hospital doctors rarely obtain authorization for services such as vaccinations, which outpatient doctors (usually family physicians) are primarily responsible for providing [21, 22]. Although the specialized outpatient clinic provides post‐transplant care, which many patients also regard as their “medical home” [13], patients must therefore be sent to the family physicians for vaccinations. As there is currently no central vaccination register or electronic vaccination card in Germany [23, 24], physicians in specialized outpatient clinics are often unaware of their patients’ vaccination status unless they actively obtain the information from the patient. As they do not administer vaccinations themselves, they may also be less aware of the need for immunizations. Additionally, previous surveys have shown that family physicians are hesitant to vaccinate vulnerable groups like organ transplant recipients [12, 13].

Taking these aspects into account and based on the results of our first intervention study, we compared the effectiveness of a three‐arm randomized intervention on vaccination rates in LT recipients, by measuring the improvement of the vaccination.

## 2 Methods

### 2.1 Procedures and Measures

A three‐arm intervention study design was used. The well‐known cohort of 401 LT recipients [9, 18], which has been previously described, was randomly divided into three groups using a randomization table, the allocation was not concealed: (1) patient intervention, (2) family physician intervention, and (3) combined patient and family physician intervention. Eighteen patients (4.5%) died during the observation period, and 94 patients (23.4%) did not provide their vaccination documentation (several unsuccessful reminders to bring the vaccination card, relocation, discontinuation of study participation), resulting in a final study cohort of 289 patients.

The intervention consisted of a yellow information letter, which was personalized and sent to either the patient (1), the family physician (2), or both (3) in December 2016. The final version of the information letters can be found in the supplement. In intervention (1) the letters were sent to the patients’ personal addresses, stressing the importance of a complete vaccination status and encouraging the patient to visit their family physician for a check of the vaccination status. In intervention (2) the letter to the family physicians (office address, patient mentioned by name) emphasized the updated recommendation for sequential pneumococcal vaccination (first conjugate vaccine, then polysaccharide vaccine) for immunocompromised patients and asked for all missing vaccinations to be completed according to the *Standing Commission on Vaccination (STIKO, Ständige Impfkommission)* recommendations at the time of intervention [25].

The vaccination status was reviewed using the patients’ vaccination documents, which were copied during routine appointments in the LT outpatient clinic in 2018 and 2019. Patients who failed to present a vaccination card during this period were asked to bring their vaccination card in 2021/2022. For those who stated that they no longer had a vaccination card, the family physician was contacted. The vaccination history was entered into a pseudonymized dataset.

The prevalence rates for the vaccinations recommended for organ transplant recipients according to the *STIKO* recommendations of 2016 and a special edition of 2005 were calculated [26, 27]. Therefore only complete vaccination schedules were included, for example, for a patient to be completely vaccinated against tetanus a complete basic immunization (3 vaccinations) and a booster in the last 10 years was necessary. Standard vaccinations (e.g., tetanus, diphtheria, pertussis, polio) are those recommended by the *STIKO* for everyone, regardless of whether a chronic underlying disease is present. Indicated vaccinations (e.g., pneumococci, influenza) are for people with an individual (non‐work‐related) increased risk of exposure, disease or complications, such as LT recipients [27]. The following vaccinations were recommended by the *STIKO* for LT recipients during the study period: pneumococci, influenza (of the current season), hepatitis A and hepatitis B. As the administration of live vaccines is contraindicated after organ transplantation, such vaccinations (e.g., measles‐mumps‐rubella) were not recommended [26, 27].

For all three groups, prevalence rates for each vaccination were quantified for 2016 (record date until 31 December 2016) and 2018 (record date until 31 December 2018). As the intervention took place in December 2016, during the 2016/2017 influenza vaccination season, the influenza vaccination rates for the 2015/2016 season were used as baseline and compared to the 2017/2018 season.

The prevalence rates for each single vaccination were used. Standard vaccines are mainly available as combination vaccines, which the STIKO primarily recommends due to their benefits [27]. The present study revealed that combination vaccines were the most commonly used type of vaccine, whereas single vaccines such as tetanus and hepatitis A were used less frequently (frequency of administration of a single vaccine in 2016 and 2018: tetanus: 10.9%, 9.5%; polio: 37.9%, 37.8%; hepatitis A: 24.0%, 26.7%; hepatitis B: 40.1%, 39.4%). As there are many different combination vaccines, we have decided to list and calculate the changes of the components of each vaccine separately, rather than looking at the doses administered.

The three quality indicators were defined according to the recommendations of the *STIKO*. These quality indicators had been used in the 2014 baseline study [9] and in the previous intervention in 2014–2016 [18]. They were adapted to the prevailing STIKO recommendations during the study [25, 26, 27]:

1. “Standard vaccinations completed”: The patient has a complete vaccination status for all vaccinations recommended for the general population, including three basic immunization vaccinations against tetanus, diphtheria, and polio, and booster vaccinations against tetanus and diphtheria every 10 years, as well as one booster against pertussis in adulthood and one booster against polio.
2. “Indicated vaccinations completed”: Due to drug immunosuppression after LT, the *STIKO* recommends at least two hepatitis A and three hepatitis B vaccinations and one influenza vaccination in each season, in addition a sequential vaccination against pneumococci (first 13‐valent conjugate vaccine [PCV 13], then 23‐valent polysaccharide vaccine [PPSV23]). For this study, those who first received PPSV 23 and then PCV 13 were also included.
3. “All vaccinations completed”: Patients who had completed all standard and all indicated vaccinations met this quality indicator.

### 2.2 Statistical Analyses

McNemar's test was used to assess the overall effect of the intervention by comparing the prevalence rate of each vaccination individually between 2016 and 2018 independently of the intervention group; to account for one‐sided testing, *p* values have been adjusted accordingly [28, 29]. Logistic regression was performed to calculate the effect of the independent variable *intervention* (letter to patient, letter to family physician, or letter to patient and family physician) on the dependent dichotomous variable *2018 vaccination status* for each vaccination. The vaccination status (vaccinated yes/no) was added to the model as an additional independent variable. This method was chosen to account for the baseline differences in vaccination rates. Previous studies showed a benefit of an approach using regression methods rather than a comparison of comparing pre‐/post‐intervention differences [30]. The lowest‐threshold intervention “letter to patient” was used as the reference group. Since the vaccination against pneumococci was explicitly mentioned in the letter to the family physician, we analyzed the implementation of this vaccination recommendation separately.

All analyses were performed using R version 4.2.2 (Foundation for Statistical Computing, Vienna, Austria) [31].

All participants received written information and signed informed consent forms. Ethical approval was obtained from the Ethics Committee of the Medical Faculty of the University of Duisburg‐Essen [reference number: 13‐5633‐BO].

## 3 Results

### 3.1 Study Population

Of the 289 patients included in the final analysis, 121 (42.0%) were female and 168 (58.0%) were male. The mean age of the patients at the beginning of the intervention was 56.3 years (range: 20.8 to 87.5 years). On average, nine years (mean: 9.1 years, SD: 6.31 years, min 2 years, max 32 years) had elapsed from the start of this intervention to liver transplantation. The most common etiologies of cirrhosis pre‐transplant were either infectious due to hepatitis B or C (28.0%), alcohol‐related (26.0%), or autoimmune (19.0%). The patient characteristics of the three intervention groups are shown in Table 1.

**TABLE 1:** Description of study population.

	Overall, *n* = 289	Letter to patient, *n* = 98	Letter to family physician, *n* = 101	Letter to patient & family physician, *n* = 90
**Gender**				
Female	121 (42%)	38 (39%)	42 (42%)	41 (46%)
Male	168 (58%)	60 (61%)	59 (58%)	49 (54%)
**Age** (mean, SD)	56 (13)	56 (14)	57 (13)	56 (13)
**Etiology**				
Alcohol‐related	76 (26%)	24 (24%)	26 (26%)	26 (29%)
Infectious (hepatitis B/C)	80 (28%)	29 (30%)	26 (26%)	25 (28%)
Autoimmune	54 (19%)	20 (20%)	19 (19%)	15 (17%)
Congenital/hereditary	27 (9.3%)	10 (10%)	10 (9.9%)	7 (7.8%)
Vascular	6 (2.1%)	1 (1.0%)	2 (2.0%)	3 (3.3%)
Tumors	15 (5.2%)	2 (2.0%)	6 (5.9%)	7 (7.8%)
Cysts and abscesses	6 (2.1%)	3 (3.1%)	2 (2.0%)	1 (1.1%)
Unknown	25 (8.7%)	9 (9.2%)	10 (9.9%)	6 (6.7%)

### 3.2 Overall Effect of the Intervention

**FIGURE 1:**
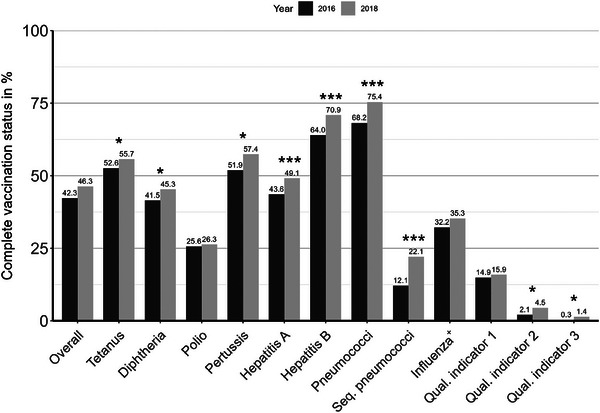
Vaccination rates for the overall intervention at baseline (12/31/2016) and follow‐up (12/31/2018). ^+^Influenza the vaccination rates of season 2015/16 were used as baseline and those of season 2017/18 as follow‐up. *p < 0.05; ***p < 0.001 in McNemar's tests.

Vaccination coverage rates at baseline and follow‐up across all interventions are shown in Figure 1. An increase from baseline to follow‐up was observed for all vaccinations (+4%), in particular for pertussis (51.9%–57.4%), hepatitis A (43.6%–49.1%), hepatitis B (64.0%–70.9%), and pneumococci (68.2%–75.4%). The largest increase of 10% was found for the sequential pneumococcal vaccination (12.1%–22.1%). The intervention as a whole had a significant effect on the following vaccinations: tetanus (McNemar's *χ* ^2^ = 3.522, *p*  =  0.031), diphtheria (McNemar's *χ* ^2^ = 5.261, *p* = 0.011), pertussis (McNemar's *χ* ^2^ = 7.529, *p* = 0.003), hepatitis A (McNemar's *χ* ^2^ = 16, *p* < 0.001), hepatitis B (McNemar's *χ* ^2^ = 20, *p* < 0.001), pneumococci (McNemar's *χ* ^2^ = 13.364, *p* < 0.001), and sequential pneumococcal vaccination (McNemar's *χ* ^2^ = 29, *p* < 0.001). There was no significant effect of the intervention on the following vaccinations: influenza (McNemar's *χ* ^2^ = 1.246, *p* = 0.132), and polio (McNemar's *χ* ^2^ = 1.000, *p* = 0.159).

Concerning quality indicator 1 “complete standard vaccination,” 43 patients (14.9%) had a complete vaccination status at baseline, increasing to 46 patients (15.9%) at follow‐up, which was not significant (McNemar's *χ* ^2^ = 0.692, *p* = 0.203). Of all patients, 2.1% (*n* = 6) had all vaccinations for quality indicator 2 “indicated vaccinations completed” at baseline, with a significant increase to 4.5% (*n* = 13) at follow‐up in 2018 (McNemar's *χ* ^2^ = 4.455, *p* = 0.018). Concerning quality indicator 3, 0.3% of the patients had “all vaccinations completed” in 2016, increasing significantly to 1.4% in 2018 (McNemar's *χ* ^2^ = 3.000, *p* = 0.042).

### 3.3 Effect of the Different Interventions

**FIGURE 2:**
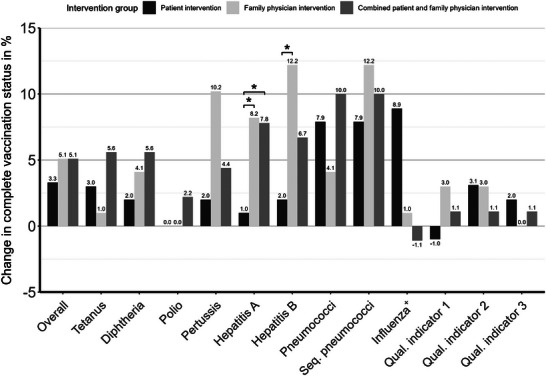
Change in vaccination rates for the three interventions at baseline (12/31/2016) and follow‐up (12/31/2018). ^+^Influenza the vaccination rates of season 2015/16 were used as baseline and those of season 2017/18 as follow‐up.

Figure 2 shows the effect on the vaccination rates for each intervention group separately.

Intervention 1 (letter to patients) showed the highest increase for the vaccination rate of influenza from baseline to follow‐up (+8.9%). In intervention 2 (letter to family physician), the increase in vaccination rate from baseline to follow‐up was highest for hepatitis B (+12.2%), and sequential pneumococci (+12.2%), followed by pertussis (+10.2%) and hepatitis A (+8.2%). For tetanus (+5.6%), diphtheria (+5.6%), polio (+2.2%), and pneumococci (+10.0%), the increase from baseline to follow‐up was highest for intervention 3 (letter to family physician and patient). Concerning the quality indicators there were no significant differences; describing Figure 2 quality indicator 1 showed the highest increase (+3.0%) for intervention 2 (letter to family physician). Quality indicator 2 showed the highest increase for intervention 1 (letter to patient) (+3.1%) and for intervention 2 (letter to family physician) (+3.0%). Quality indicator 3 showed the highest increase for intervention 1 (letter to patient) (+2.0%). Intervention 3 (letter to family physician and patient) showed an equal increase of 1.1% for all three quality indicators. More detailed information is displayed in Figure 2.

The results of the regression analyses, which compared the different types of intervention, are presented in Table 2.

**TABLE 2:** Results of the regression analyses comparing different types of intervention.

	Logistic regression					
	Letter to family physician^a^			Letter to patient and family physician^a^		
	Odds ratio	95% CI^b^	*p* value	Odds ratio	95% CI^b^	*p* value
**Vaccine**						
Tetanus	0.68	0.23, 1.98	0.5	1.38	0.48, 4.05	0.6
Diphtheria	1.15	0.40, 3.41	0.8	1.32	0.45, 3.98	0.6
Polio	1.09	0.04, 30.2	>0.9	5.22	0.43, 181	0.2
Pertussis	1.99	0.81, 5.05	0.14	1.07	0.42, 2.76	0.9
Hepatitis A	9.91	1.73, 187	0.034^*^	9.07	1.53, 173	0.043^*^
Hepatitis B	9.13	2.23, 62.3	0.006^*^	4.04	0.85, 29.1	0.10
Pneumococci	0.57	0.21, 1.52	0.3	0.97	0.37, 2.56	>0.9
Sequential pneumococci	1.50	0.59, 4.02	0.4	1.27	0.46, 3.56	0.6
Influenza^c^	0.94	0.47, 1.87	0.9	0.49	0.23, 1.02	0.061
Quality indicator 1	2.58	0.64, 12.3	0.2	1.77	0.41, 8.43	0.5
Quality indicator 2	1.06	0.24, 4.90	>0.9	0.71	0.12, 3.62	0.7
Quality indicator 3	0.00	—^d^	>0.9	0.55	0.03, 5.79	0.6

^a^ Reference: letter to patient.

^b^ CI = confidence interval.

^c^ Reference influenza season 2015/16 to 2017/18.

^d^ No patients fulfilled the criteria for quality indicator 3 for 2016 or 2018.

* *p* < 0.05.

** *p* < 0.001.

Vaccination with hepatitis A was more likely to be completed when the family physician and the patient received the letter compared to when only the patient did (OR  =  9.07, *p*  =  0.043, with a 95% CI [1.53, 173]). The intervention group with only the family physician receiving the letter showed an odds ratio of 9.91 (*p* = 0.034, 95% CI [1.73, 187]) for hepatitis A virus vaccination. The same was observed for hepatitis B, with the chance of the patient being vaccinated being higher at follow‐up in 2018 when the family physician and the patient received the letter than when only the patient did (OR = 4.04, *p* = 0.1, 95% CI [0.85, 29.1]). An odds ratio of 9.13 was observed for the intervention group in which only the family physician received the letter (*p* = 0.006, 95% CI [2.23, 62.3]).

The results of the logistic regressions calculated for the three quality indicators are displayed in Table 2. The results were not significant.

## 4 Discussion

This comparison of three information strategies (letter to the patients themselves, their treating physician, or both) to improve the immunization status of LT recipients showed higher vaccination rates when family physicians were addressed or when both family physicians and patients were informed simultaneously compared to when addressing patients alone. Overall, the intervention showed significant improvements in vaccination rates for tetanus, pertussis, diphtheria, hepatitis A and B, pneumococci and the sequential pneumococci vaccination, regardless of who was contacted. Looking at the different kinds of intervention separately, a significant advantage of intervention 2 (letter to family physician) and 3 (letter to patient and family physician) was demonstrated, especially for the vaccinations against hepatitis A and B, compared to intervention 1 (letter to patients). Overall, the quality indicators showed only a slight (increase of 1.0% for quality indicator 1) but significant improvement (2.4% for quality indicator 2, and 1.1% for quality indicator 3) by the intervention.

In general, the interventions were effective. In particular, involving the family physician, whether simultaneously with the patient or alone, was helpful. While informing patients alone also resulted in improved vaccination rates, it did so to a lesser extent.

In line with other studies [14, 32], a combination of patient and provider reminders appears to be more effective in increasing vaccination rates than patient reminders alone. Like in the study of Humiston et al., which examined the effect of patient reminders in combination with provider reminders, we also used a bright yellow letter for both family physicians and patients. Humiston et al. were thus able to achieve a significant increase in the influenza vaccination rate among older (non‐organ transplant) subjects compared to the control group (64.0% vs. 22.0%) [32]. Although our study did not achieve such a clear effect on vaccination rates, particularly on those for influenza, it showed that targeting the family physician to complete the vaccination status of the patients named in the letters was superior. This was particularly obvious for hepatitis vaccinations: the odds of achieving a sufficient vaccination status for hepatitis A and B was 10 and 9 times higher, respectively, when contacting the family physician compared to contacting the patient alone. One possible explanation is that doctors can easily contact patients by telephone via their medical assistants to make an appointment. In some cases, it may be more challenging for patients to contact their family physician for the vaccination, especially if the practice is difficult to reach by telephone. Another explanation is a lack of motivation among LT recipients to visit their family physician for vaccinations, as they already have a high number of regular appointments at specialized outpatient clinics. This aspect was already mentioned in a study conducted by Feldman et al. in interviews with parents of children listed for transplantation [13]. As long as vaccinations cannot be administered routinely in specialized outpatient clinics in Germany and there are no affiliated special vaccination clinics/facilities for immunosuppressed persons such as organ transplant patients [33], there is a need for greater involvement of family physicians to increase vaccination rates among particularly vulnerable patient groups. Therefore, vaccination recommendations should be regularly included in outpatient letters, the more specific the better. Sanftenberg et al. noted that most family physicians are willing to implement such vaccination recommendations [17]. However, this requires training of staff in specialized outpatient clinics, both in terms of organization, for example, regularly requesting and inspecting patients’ vaccination cards, and in terms of including standard and indicated vaccinations in the context of specific recommendations.

In a study conducted by Schulte et al., influenza vaccination rates among kidney transplant patients increased by 8.3% when doctors at the kidney transplant outpatient clinic at the University Medical Center Schleswig‐Holstein in Kiel encouraged their patients to get vaccinated. A vaccination appeal to outpatient nephrologists had no effect on influenza vaccination coverage rates [34]. While both the family physician intervention and the intervention with information to the family physician and the patient were generally superior to the patient‐centered intervention alone in our study, this was not the case for influenza vaccinations. There were no significant differences for the different interventions for the influenza vaccination; however, patient contact alone seemed to be more beneficial compared to the other two interventions. This could be due to the fact that physicians consider the effectiveness of the vaccine to be too low [13], or due to the timing of the intervention. The letters were sent in December 2016, at a time when most people receiving an influenza vaccination had already been vaccinated for the 2016/2017 season [35]. By the time of vaccination for the 2017/2018 season, too much time may have passed since the intervention and therefore the effect may have faded [36].

As part of the intervention, the new STIKO recommendation for sequential pneumococcal vaccination in immunosuppressed persons [25] was explicitly addressed in the letters to the family physicians. Overall, the intervention increased the pneumococcal vaccination rate by 7.2%. In particular, the letter to both the family physician and the patient was superior to informing the family physician or the patient alone. The share of patients with sequential vaccination also increased by 12.2%, especially in the family physician‐letter intervention group. Most patients received the 23‐valent polysaccharide vaccine first and then the 13‐valent conjugate vaccine, since many of these patients had already been vaccinated with the polysaccharide vaccine prior to the new STIKO recommendation. Since 2023, the STIKO recommends only the 20‐valent pneumococcal conjugate vaccine, even for immunocompromised adults. The previous recommendation of sequential vaccination of particularly vulnerable adult patient groups, such as LT recipients, has since been dropped [37]. This may lead to greater acceptance of the pneumococcal vaccination by physicians and patients and increase vaccination rates in the future.

For this study we concentrated on the prevalence rates for each single vaccination. The absence of a single diphtheria vaccine, coupled with the necessity of administering combination vaccines that include tetanus, elucidates the nearly equivalent rise in vaccination rates between 2016 and 2018 (tetanus: 3.1%, diphtheria: 3.8%). A comparable situation is observed for pertussis, as vaccination is only possible in combination with tetanus and diphtheria. However, the increase in the vaccination rate is slightly higher (5.5%) because, in contrast to tetanus‐diphtheria, a single vaccination is sufficient to attain full vaccination status [27]. The polio vaccination rate remained relatively stable between 2016 and 2018, despite the availability of monovalent vaccines. This finding suggests that the provision of a monovalent vaccine does not invariably result in increased vaccination rates. A slightly higher increase in vaccination rates was observed for hepatitis B (6.9%) compared to hepatitis A (5.5%). This increase is consistent with the increased administration of single hepatitis B vaccines and with the different definitions of complete vaccination protection for hepatitis A and B [27].

### 4.1 Comparison of Vaccination Rates With Those of the Baseline Study Cohort

Despite the limited comparability of vaccination rates of the 2018 LT cohort with those of the 2014 baseline survey [9] due to the high dropout rate over the years, there are indications of an increase in vaccination rates after the two interventions, for example: hepatitis A (course completed): 23% in 2014 compared to 49.1% in 2018; hepatitis B (course completed): 39.6% to 70.9%, pneumococcal vaccination (at least one vaccination during lifetime): 59.7% to 75.4%. The influenza vaccination rate increased by 11% overall over the years (from 24% to 35%) with only a small increase achieved with the last intervention. The two interventions might have already reached those who wanted to be vaccinated. The second intervention led to an only minor improvement in the quality indicators, suggesting that a written intervention is not sufficient to reach the very high goal of a complete vaccination status for all standard and indication vaccinations. To achieve this, barriers to cross‐sectoral care need to be broken down and patients should either be vaccinated directly in specialized outpatient clinics to simplify the process for the patients, or low‐threshold communication channels need to be established between family physicians and specialized outpatient clinics [1]. Feldmann et al. explored the possibilities of a transplant digital health tool [1] that could be implemented with the mandatory introduction of electronic patient records in Germany in 2025 [38]. By storing vaccination card data in the electronic patient record, all treating physicians and patients (the latter via a mobile app) would be able to access and view this data. Specialized outpatient clinics could provide advice on the appropriate timing of vaccination, contraindications to certain immunosuppressant drugs, and vaccination recommendations. A reminder system could be used to regularly remind both patients and family physicians of necessary vaccinations. In the future, low‐threshold ad‐hoc communication channels will be facilitated by so‐called TI (telematics infrastructure) messengers, quickly clarifying queries from family physicians by specialized outpatient clinics.

### 4.2 Strengths and Limitations

The strength of the study is the randomization of patients into the three intervention groups, which resulted in balanced groups characteristics. However, no control group was used in the study due to ethical reasons. Another strength is that the study is a real‐life quality improvement project for a high‐risk group within the German healthcare system. Furthermore, an evaluation of the components of vaccines, as opposed to combination vaccines, enables a more precise assessment of vaccine protection against specific diseases. This approach facilitates enhanced comparability with national and international vaccination recommendations, which frequently offer targeted recommendations for specific diseases.

In terms of limitations, it is possible that vaccinations may have been given but not recorded in the vaccination card, which is common especially for influenza vaccinations. As in the two previous studies [9, 18], vaccination coverage rates were analyzed from vaccination records. A number of 112 patients (27.9%) dropped out since 2016 due to various reasons such as missing vaccination documentation, relocation, discontinuation of study participation and death, possibly effecting the outcome. However, it cannot be ruled out that patients who were particularly vulnerable, unwilling, or eager to be vaccinated were excluded, which could have led to selection bias. The comparability of the two interventions is also limited by the high dropout rate, which additionally results in a loss of statistical power.

This intervention had no stronger effect on vaccination rates than the first intervention [18] and in some cases it was even less effective. This could be due to the fact that the same sample had already received comparable interventions and was less susceptible to this renewed call for vaccination.

## 5 Conclusion

In conclusion, this study shows the particular importance of family physicians in improving vaccination rates in LT recipients in Germany. Establishing low‐threshold communication channels, for example, via the electronic patient record or TI messengers, could improve collaboration between physicians in specialized outpatient clinics and family physicians. The concerns of some family physicians about vaccinating these vulnerable patient groups could be allayed through these channels, improving the vaccination readiness of the family physicians and thereby improving the vaccination status of the patients.

## Author Contributions

Dorothea Dehnen collected the data, supported statistical analysis and data interpretation, prepared the first draft of the manuscript and revised it in communication with the co‐authors. Maia Milzkott collected the data, prepared and revised the manuscript. Anette Graute and Benjamin Borchardt performed statistical analysis and data interpretation. Katja Dehnen supported statistical analysis and data interpretation, prepared and revised the manuscript. Anna Herwig developed the study concept, collected data. Kerstin Herzer developed the study idea and the concept, supported the data collection. Katharina Willuweit and Jassin Rashidi‐Alavijeh supported the data collection, revised the manuscript. Birgitta Weltermann developed the study idea and concept, supported data interpretation, and revised the manuscript.

## Conflicts of Interest

The authors declare no conflicts of interest.

## Data Availability

The data that support the findings of this study are available on request from the corresponding author. The data are not publicly available due to privacy or ethical restrictions.
